# Case report: Amisulpride therapy induced reversible elevation of creatine kinase-MB and bradycardia in schizophrenia

**DOI:** 10.3389/fpsyt.2022.1037738

**Published:** 2022-12-14

**Authors:** Ze-Rui Hu, Zhen-Zhen Yang, Xu-Bo Wang, Hong-Shuo Chu, Chuan-Xin Liu

**Affiliations:** ^1^Department of Psychiatry, School of Mental Health, Jining Medical University, Jining, China; ^2^Department of Psychiatry, Shandong Daizhuang Hospital, Jining, Shandong Province, China; ^3^Department of Cardiology, Jining No.1 Hospital, Jining, Shandong Province, China

**Keywords:** schizophrenia, serum myocardial enzyme spectrum, amisulpride, bradycardia, case report

## Abstract

**Introduction:**

Schizophrenia is regarded as one of the most severe, disabling, and costly mental illnesses. Hence, early effective prevention and treatment are critical to the prognosis of patients. Amisulpride, a first-line atypical antipsychotic medication that acts as a blocker of the D2 and D3 dopamine receptors, is used in varying doses for the treatment of both positive and negative symptoms of schizophrenia. Reversible amisulpride-induced elevation of the myocardial enzyme spectrum with bradycardia is a rare condition.

**Case presentation:**

We report a 26-year-old patient diagnosed with first-episode schizophrenia. This patient was treated with amisulpride (400 mg/d), but no clinical benefits were obtained. Meanwhile, amisulpride caused elevation of the myocardial enzyme spectrum with asymptomatic bradycardia. After stopping the medication, these parameters normalized.

**Conclusion:**

We described a rare side reaction of amisulpride. Psychiatrists should take this side effect seriously in the clinical setting. The mechanism of this adverse reaction warrants further investigation and debate. When this side effect occurs during treatment, reducing the dosage of amisulpride and subsequently discontinuing medication, along with monitoring the electrocardiogram and serum myocardial enzymes, may be the most appropriate treatment protocol.

## Introduction

Schizophrenia is identified as one of the most severe, incapacitating, and expensive mental illnesses, affecting up to 1% of the population (1). Schizophrenia is characterized by a cluster of positive or negative symptoms and deterioration in social, work, or interpersonal relationships. As a globally critical health concern, it still needs more research funding to investigate its causes, prevention, and treatment. Amisulpride, an atypical antipsychotic, selectively blocks the D2/D3 receptor, making it a first-line of medication for schizophrenia (2, 3). It has been proven that amisulpride has a significant effect on the positive symptoms of schizophrenia and a modest efficacy in treating negative symptoms (4–6). For the safety of the medication, it is essential to monitor the adverse effects of amisulpride, such as hyperprolactinemia, extrapyramidal side effects, and QT prolongation (6, 7). In this case, we report a rare side reaction during treatment, the reversible amisulpride-induced elevation of CK-MB and bradycardia.

## Case presentation

A 26-year-old male diagnosed with first-episode schizophrenia had predominant negative symptoms for nearly 6 years without anti-psychotic medication because of stigma and family neglect of his symptoms until his first visit to our hospital in February 2022. The patient, from a rural family of middle socio-economic status, had been unemployed since he graduated from high school. Due to lack of insight, the patient did not seek any medical care by himself. When the patient's family realized that the patient had psychiatric symptoms, they brought the patient to our hospital for treatment. The main clinical manifestations of this patient are affective blunting, alogia, avolition, attentional impairment, and delusion of reference. The major differential diagnosis was major depression. The diagnosis of major depressive disorder was excluded because this patient with markedly impaired social functioning had no obvious depressed mood or anhedonia, and his clinical course was persistent over 6 years. Physical examination, electrocardiogram, and routine blood tests were performed on admission with no abnormal findings. After admission, routine examinations were applied to rule out organic disease and improve clinical medication safety. The cranial computed tomography and electroencephalogram did not reveal any abnormalities. Furthermore, laboratory examination showed normal renal, liver, and thyroid functions. Serum electrolytes, blood glucose and lipid levels, and serum myocardial enzymes were also within normal limits. The patient was treated with amisulpride 400 mg/d. After 2 weeks of treatment, routine serum laboratory tests showed that high creatine kinase of MB (CK-MB) (104 U/L), serum creatine kinase (CK) (13660 U/L), lactate dehydrogenase (LDH) (387 U/L), and myoglobin (Mb) (360.6 ug/L) with normal levels of cardiac troponin I (cTnI). Furthermore, the patient had significantly high levels of aspartate aminotransferase (AST) (146 U/L) and alanine aminotransferase (ALT) (71 U/L). And when the ECG revealed bradycardia (52 beats per minute), the Holter monitor showed high-frequency heart rate variability (HF-HRV) (2081.01 ms^2^) and low-frequency heart rate variability (LF-HRV) (1542.12 ms^2^) increased significantly compared to the baseline HF-HRV (1649.44 ms^2^) and LF-HRV (1325.24 ms^2^), while the LF/HF ratio (0.74) decreased than the baseline (0.80) (Table 1). There were no reports of discomfort, such as chest pain or myalgia, and echocardiography revealed no abnormalities. Moreover, the patient had no medical history or family history of cardiovascular disease. Because of the poor therapeutic efficacy, bradycardia, and CK-MB elevation, the medicine was reduced (200 mg/d) on the 17th day after admission and discontinued on the 21st day after admission. After that, the level of the myocardial enzyme spectrum decreased and eventually recovered to the normal range. Furthermore, due to low overall efficacy and the adverse effect of amisulpride, the treatment scheme was modified to include aripiprazole medication. After the aripiprazole medication, this patient's serum myocardial enzymes and heart rate were within the normal range (Table 1). The negative symptoms of this patient improved significantly and remained stable.

**Table 1:** Timeline of relative indicator.

	**Day after hospitalization**					
	**1**	**17** **(amisulpride dose reduction after detection of CK elevation)**	**21** **(amisulpride discontinuation)**	**28**	**42**	**57**
CK (U/L)	230	13660	1484	694	183	180
CK-MB (U/L)	20	110	33	22	15	20
cTnI (U/L)	0.42	0.15	0.1	0.2	-	0.16
MB (U/L)	128.4	360.6	112	83	-	38.5
HR (bpm)	89	52	54	75	70	92
LF (ms^2^)	1325.24	-	1542.12	1244.5	-	-
HF (ms^2^)	1649.44	-	2080.01	1391.21	-	-
LF/HF ratio	0.8	-	0.74	0.89	-	-

CK, creatine kinase; CK-MB, creatine kinase-MB; cTnI, cardiac troponin I; HR, heart rate; LF, low frequency; HF, high frequency; LF/HF ratio, the ratio of low frequency and high frequency; bpm, beats per minute.

Reference value: CK (50–310 U/L); CK-MB (0–25 U/L); cTnI (0–1.68U/L); MB (5–100 μg/L); HR (60–100 bpm); LF (1,170 ± 416 ms^2^); HF (975 ± 203 ms^2^); LF/HF ratio (1.5–2.0).

## Discussion

It is well-known that amisulpride has several cardiac side effects. A preclinical study showed that amisulpride induced cardiac lesions in rabbits, such as necrosis and deformation of the nuclei (8). Qu et al. (9) indicated that amisulpride could affect cardiac functions, and the changed levels of LDH and CK were related to the plasma concentration of amisulpride. According to a recent meta-analysis, the common cardiac side effect of amisulpride is QTc prolongation (7). Moreover, He et al. (10) illustrated that amisulpride performed strong cardiotoxicities, such as cardiomyopathy, QTc prolongation/Torsade de Pointes and cardiac arrhythmia, among ten antipsychotics (clozapine, olanzapine, etc.) based on the analysis of side effect records on the Adverse Event Reporting System database. And there were several case reports that demonstrated amisulpride could induce bradycardia (11, 12) and hypertension (13) as well as elevation of CK (14, 15).

We experienced a rare case of a patient who had a reversible elevation of CK-MB accompanied by bradycardia. Naranjo Scale, a method designed by Naranjo et al. for estimating the probability of adverse drug reactions, was applied to assess the relationship between amisulpride and this rare side reaction (16). As the Naranjo Scale score was 6, this side effect was probably related to amisulpride. This case has several explanations, but none fully explain this phenomenon. First, CK-MB, an isozyme of CK, mainly exists in cardiac muscle. And there are trace amounts of CK-MB in skeletal muscle (Table 2). CK-MB and cTnI as cardiac markers assist in diagnoses of myocardial infarction (17). Considering the normal level of cTnI, the evidence of myocardial injury is insufficient (18). However, because of the CK-MB elevation, the functional abnormality of the cardiocyte remains conceivable. Secondly, the neuroleptic malignant syndrome is excluded due to the absence of related symptoms including fever and rigid muscles (19). Thirdly, based on the high level of CK (13,660 U/L), a diagnosis of asymptomatic rhabdomyolysis should be considered, though without myoglobinuria, electrolyte abnormalities, or acute kidney injury. The exact pathophysiology of the antipsychotic-induced CK-MB and CK elevation is not known. It may be connected with the low-effect central action on dopamine receptors in the striatum or direct toxic effects on the sarcolemma (20, 21). The increased HF-HRV and decreased LF/HF ratio suggested that the activation of the brain's muscarinic cholinergic system by amisulpride may have a role in bradycardia (Table 2) (22). The patient rejected additional testing, electromyography, and musculoskeletal biopsy. Therefore, the diagnosis and differential diagnosis is uncertain. Because the amisulpride drug concentration test is not available in our hospital, it is difficult to determine if there is a drug overdose without the patient's plasma level of amisulpride. There appears to be no literature reporting the amisulpride-induced elevation of CK-MB with asymptomatic bradycardia. To our knowledge, there is no report of this adverse reaction on pharmacovigilance databases such as WHO's VigiAccess (23) and Side Effect Resouce 4.1 (SIDER 4.1) (24).

**Table 2:** Information on the relative indicator.

	**Locations**	**Functions/Definitions**	**Clinical implications of elevated levels**
Creatine kinase (CK)	Skeletal muscle, brain, heart, smooth muscle, etc.	CK catalyzes conversion of creatine and uses adenosine triphosphate (ATP) to create phosphocreatine (PCr) and adenosine diphosphate (ADP).	Acute myocardial infarction, myocarditis, rhabdomyolysis, or neuroleptic malignant syndrome, etc.
Creatine kinase-MB (CK-MB)	Cardiac muscle (main), skeletal muscle (trace amounts).	CK-MB catalyzes conversion of creatine and uses adenosine triphosphate (ATP) to create phosphocreatine (PCr) and adenosine diphosphate (ADP).	Acute myocardial infarction, myocardial ischemia, or myocarditis, severe skeletal muscle damage, etc.
Cardic troponin I (cTnI)	Cardiac muscle	cTnI binds to actin in thin myofilaments.	Cardiac marker with high affinity for acute myocardial infarction.
Myoglobin (MB)	Cardiac and skeletal muscle	MB, an oxygen-binding protein, stores oxygen.	Acute myocardial injury, rhabdomyolysis, kidney failure, etc.
Lactate dehydrogenase (LDH)	Nearly all living cells	LDH catalyzes the interconversion of pyruvate to lactate.	Acute myocardial infarction, hepatitis, acute kidney disease, acute pancreatitis, etc.
Aspartate aminotransferase (AST)	Liver, heart, skeletal muscle, kidney	AST catalyzes the interconversion of aspartate and α-ketoglutarate to oxaloacetate and glutamate.	Hepatitis, acute myocardial infarction, acute pancreatitis, etc.
Alanine aminotransferase (ALT)	Liver, heart, skeletal muscle, kidney	ALT catalyzes the interconversion of L-alanine and α-ketoglutarate to pyruvate and L-glutamate.	Hepatitis, acute myocardial infarction, cancer, diabetes, etc.
Low frequency (LF)	–	The number of N-N intervals that match the low frequency band (0.04–0.15 Hz) in Holter.	LF reflects sympathetic modulations.
High frequency (HF)	–	The number of N-N intervals that match the high frequency band (0.15–0.4 Hz) in Holter.	HF reflects the vagal modulations.
LF/HF ratio	–	The ratio of LF to HF in Holter.	LF/HF reflects the sympathovagal balance or sympathetic modulations.

N-N intervals, normal-to-normal intervals (all intervals between adjacent QRS complexes resulting from sinus node depolarization).

The mechanism of this adverse reaction deserves more investigation and discussion. This rare side effect should be considered in amisulpride intervention. When this side effect occurs during treatment, reducing the dosage of amisulpride and subsequently discontinuing medication, along with monitoring the electrocardiogram and serum myocardial enzymes, may be the most appropriate treatment protocol.

## Data availability statement

The original contributions presented in the study are included in the article/supplementary material, further inquiries can be directed to the corresponding author.

## Ethics statement

Ethical review and approval was not required for the current study in accordance with the local legislation and institutional requirements. The patients/participants provided their written informed consent to participate in this study. Written informed consent was obtained from the individual(s) for the publication of any potentially identifiable images or data included in this article.

## Author contributions

Z-RH: conceptualization, data curation, and writing—original draft preparation. Z-ZY: investigation and formal analysis. X-BW: data curation and visualization. H-SC: writing—review and editing. C-XL: supervision. All authors contributed to the article and approved the submitted version.
